# A *Mycobacterium tuberculosis* Beijing strain persists at high rates and extends its geographic boundaries 20 years after importation

**DOI:** 10.1038/s41598-019-40525-6

**Published:** 2019-03-18

**Authors:** Laura Pérez-Lago, María Isolina Campos-Herrero, Fernando Cañas, Rodolfo Copado, Laura Sante, Berta Pino, María Lecuona, Óscar Díez Gil, Carlos Martín, Patricia Muñoz, Darío García-de-Viedma, Sofía Samper

**Affiliations:** 1 Servicio Microbiología Clínica y Enfermedades Infecciosas, Hospital General Universitario Gregorio Marañón, Madrid, Spain; 2 Instituto de Investigación Sanitaria Gregorio Marañón, Madrid, Spain; 3 Hospital Universitario de Gran Canaria Dr. Negrín, Las Palmas de Gran Canaria, Gran Canaria, Spain; 4 Hospital Universitario Insular de Gran Canaria, Las Palmas de Gran Canaria, Gran Canaria, Spain; 5 Hospital José Molina Orosa, Lanzarote, Gran Canaria, Spain; 6 Hospital Universitario de Canarias, Tenerife, Spain; 7 Hospital Na Sa de la Candelaria, Tenerife, Spain; 8 CIBER Enfermedades respiratorias, CIBERES, Madrid, Spain; 9 IIS Aragón, Zaragoza, Spain; 10 Department of Medicine, Universidad Complutense de Madrid, Madrid, Spain; 11 Instituto Aragonés de Ciencias de la Salud, Zaragoza, Spain; 12 Hospital Miguel Servet, Zaragoza, Spain

## Abstract

Transmission of Beijing *Mycobacterium tuberculosis* can be investigated based on genotypic analysis of clinical isolates. A Beijing strain began to spread on Gran Canaria Island, Spain, at the end of the last century. In 1996, only 3 years after its importation to the island, its frequency had increased to 27.1% of all the isolates. The strain was tracked during the following years, and the most recent data obtained corresponded to 2007-8, when its presence continued to be alarming (21%). In the current study, we updated data on the distribution of this strain 20 years (2013–2014) after it was first detected on the island and extended the analysis for the first time to all the mycobacteriology laboratories covering the population of the Canary Island archipelago. Rapid updating was enabled by means of 2 different strain-specific PCRs: one targeting a peculiar feature of the strain, which was identified based on an IS*6110* copy mapping in the *Rv2180c* gene, and a newly defined strain-specific single nucleotide polymorphism, which was identified by whole-genome sequencing. The results showed that the strain has remained highly prevalent (20.90% of all isolates), has spread throughout the neighbouring islands, and has also reached high representativeness in them (11–32%).

## Introduction

The Canary Islands are an archipelago located in the Atlantic Ocean, close to the North-West African coast. The archipelago comprises 7 main islands grouped into 2 provinces—Las Palmas (Gran Canaria, Lanzarote, and Fuerteventura islands) and Santa Cruz de Tenerife (Tenerife, La Palma, La Gomera, and El Hierro islands)—which constitute an autonomous community of Spain. More than 2 million inhabitants are distributed among the most populated islands (Tenerife, Gran Canaria, Lanzarote, and Fuerteventura). The number of declared cases of tuberculosis in the Canary Islands was 162 in 2013 and 151 in 2014, with incidence at 7/100 000 inhabitants in 2013, one of the lowest incidence rates in Spain^1–3^. Las Palmas, the province closest to the African cost, presented a slightly higher incidence than Santa Cruz de Tenerife province in 2013 (8.81/100 000 vs 5.14/100 000) and in 2014 (9.61/100 000 vs 4.29/100 000). The latest official data, which are from 2016, show a decrease to 6.36/100 000 in Las Palmas, 4.28/100 000 in Santa Cruz de Tenerife, and 5.34/100 000 in the autonomous community as a whole.

Due to its rapid spread and association with numerous outbreaks, the East Asian lineage of *M*. *tuberculosis* strains has caused global concern. One major clade in this lineage, Beijing, has a well-known spoligoprofile (deleted signals from 1 to 34). Originating in the Far East, the Beijing clade spread throughout the world in several waves, and some strains have been associated with the massive spread of multidrug-resistant tuberculosis in Europe and Asia^4^. Beijing strains have been involved in large outbreaks that expanded rapidly in specific populations^5,6^.

The introduction of a Beijing strain on Gran Canaria Island and its fast spread in this community (27.1% of cases of tuberculosis) was detected in the 1990s. The index case was thought to be a refugee from Liberia diagnosed with smear-positive pulmonary tuberculosis in 1993^7^. The explosive dissemination of this strain, designated GC1237, led to it being tracked at different time-points, although no new data have become available since the most recent study (2007–2008), which was restricted to the province of Las Palmas^8^.

The current study provides an update on the presence of this successful clone and discusses 2 relevant novelties. For the first time, isolates from the whole autonomous community (including data from the four islands belonging to the second province) are analyzed. In addition, we applied a novel dual screening approach supported by 2 rapid methods based on strain-specific multiplex PCRs.

## Material and Methods

### Study sample

244 positive *M*. *tuberculosis* complex cultures (1 isolate per patient) from the 313 cases microbiologically confirmed in the 2013-4 period were available in the restrospective collection obtained from Hospital Universitario de Gran Canaria Dr. Negrín, Hospital Universitario Insular de Gran Canaria (Gran Canaria island), Hospital José Molina Orosa (Lanzarote island), Hospital Universitario de Canarias and Hospital Nª Sª de la Candelaria (Tenerife island).

The strains were subcultured in MGIT (Mycobacterial Growth Indicator Tube) or Lowenstein-Jensen medium. Five hundred microliters of inactivated culture was delivered to the University of Zaragoza. In order to fulfil ethical requirements, the isolates were identified by a code not linked to patient data.

From the inactivated material, we obtained 2 identical aliquots of 100 µl each for the following: 1) PCR targeting the IS*6110* copy in *Rv2180c*, which is specific for the GC1237 strain^8^, performed in Zaragoza; and 2) multiplex ASO-PCR based on whole genome sequencing (WGS)^9^, performed in Madrid.

### PCR targeting specific IS*6110*

**Figure 1:**
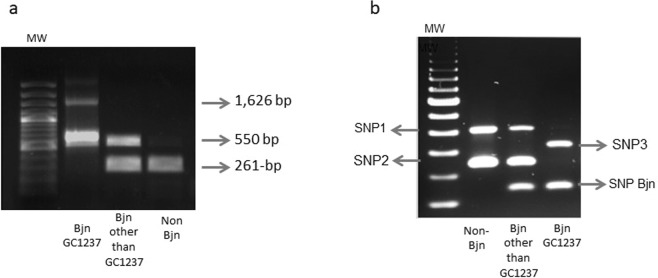
Amplification patterns expected for a Beijing strain (Bjn), the Beijing GC1237 clone and a non- Beijing strain by the IS*6110*-PCR (**a**) and ASO-PCR (**b**) approaches.

The multiplex PCR targets 2 insertion sites of IS*6110*, one located in the intergenic region *dnaA-dnaN*, which is specific for the Beijing lineage, and the other in *Rv2180*, which is specific for the Beijing-GC genotype (GC1237 clone). The results showed a pattern of 2 amplicons (1,626 bp and 550 bp) for Beijing-GC genotypes, and another 2-band pattern (550 bp and 261-bp) for any other Beijing non-GC genotypes. A single-261 bp fragment is expected for non-Beijing genotypes^8^ (Fig. 1a).

For each liquid culture, 100 µl was washed in water and heated at 90 °C for 30 minutes. The sample was then spin-dried at 13 000 rpm for 15 minutes, and 2 µl of the supernatant was used as a template for the PCR. The procedure followed a protocol described elsewhere^8^. The controls comprised purified DNAs from GC1237, a Beijing non-GC genotype strain, and H37Rv.

### Multiplex allele-specific PCR (ASO-PCR)

Design and optimization of the ASO-PCR were as previously described^9^. Briefly, we carried out WGS of 4 sequential isolates from 1 of the cases infected by the GC1237 strain and identified the strain-specific SNPs after comparing the WGS data with 2 SNP databases, one containing 219 genomes from strains circulating throughout the world and the other including 359 unique Beijing genomes. We selected 3 of these SNPs to be targeted by the ASO-PCR (*Rv2524* [C1398T], *Rv3869* [G1347C], and *Rv0926c* [G162A]) and included 1 additional SNP specific for the Beijing lineage^10^. Two of the 4 selective primers for the multiplex PCR targeted the alleles found in GC1237 (one corresponded to the Beijing marker-SNP), and the remaining 2 primers targeted the alleles expected for the non-GC1237 genotypes. Thus, 3 different amplification signatures can be obtained depending on whether the strain tested is the GC1237 genotype, a Beijing non-GC1237 genotype, or any other non-Beijing genotype (Fig. 1b). The multiplex ASO-PCR was applied directly on 5 µl from the inactivated cultures received from the University of Zaragoza.

## Results

A total of 244 inactivated cultures were collected. Results were obtained from 167 and 237 isolates after applying IS*6110*-PCR and ASO-PCR, respectively. The differences in the number of isolates with a result were mainly due to a complete lack of amplification for the IS*6110*-PCR in 1 of the deliveries, which included 77 isolates.

Hands-on time for the PCRs was around 1 hour per set of specimens sent, including PCR and electrophoresis. Final results were obtained in 2 working days.

For the 167 isolates with results for each of the 2 techniques, data were fully concordant. Seventeen Beijing isolates were not detected by IS*6110*-PCR because they were included in the delivery with no results for this technique.

A total of 53 Beijing isolates were detected; of these, 51 corresponded to Beijing-GC1237 genotype isolates. All but 2 were pan-susceptible; one isolate was mono-resistant to isoniazid, and the other was multidrug-resistant. All but 1 of the Beijing-GC genotype isolates analyzed corresponded to Spanish cases, the remaining isolate being from a Swedish case. The 2 non-GC1237 Beijing strains were also pan-susceptible and corresponded to a French patient and to a girl aged <2 years born in the Canary Islands to Moroccan parents.

The global representativeness of the Beijing-GC1237 genotype was 20.90%. The GC1237 strain was well represented on all the islands, with a wide variety between local rates, ranging from 10.41% in Tenerife to 32% in Lanzarote. The rate for Gran Canaria Island was 20.71%, and the rate for the remaining islands, La Palma and Fuerteventura, was slightly superior, 25 and 27% (Table 1).

**Table 1:** Geographic distribution in the different islands of the arhipelago and representativity of the Beijing lineage and the Beijing GC1237-outbreak strain from the data obtained applying strain-specific-PCR based strategies for the 2013-4 period.

Island	Beijing	Beijing GC	Total isolates	Percentage GC1237
Gran Canaria	31	29	140	20,71
Fuerteventura	4	4	15	26,66
Lanzarote	12	12	37	32,43
Tenerife	5	5	48	10,41
La Palma	1	1	4	25
	53	51	244	20,90

## Discussion

Tuberculosis continues to cause disease, even in countries with good health care such as Spain, which had an incidence of 10/100 000 in 2016^11^. Moreover, the figures from the study setting, the Autonomous Community of Canarias, were among the lowest incidence rates (7/100 000) for Spain in 2013.

Some lineages of *M*. *tuberculosis* complex have shown different infective and epidemiological behaviours. The Beijing lineage (included in lineage 2), which has been associated with large MDR tuberculosis outbreaks elsewhere and seems to be expanding rapidly in settings with contrasting incidence levels of the disease^5,6^. These observations have led to consider to some representatives of this lineage as candidates of higher transmissibility. The dynamics (Table 2) of a Beijing strain in Gran Canaria is remarkable: after its arrival in the 1990s via an infected migrant, it somehow managed to settle efficiently in the community, reaching rates of 21% only a few years later, consistent with the selective advantage attributed to the lineage^7^.

**Table 2:** Global TB incidence, and representativity of the Beijing lineage and the Beijing GC1237-outbreak strain since the first isolate was identified in 1993, including the data from this study (2013-4) and those from previous studies (1993–2008).

Period	Incidence rate/100 000	N isolates analyzed	N Beijing (%)	N strain GC1237 (%)	Sampled population	Reference
1993	28.5	179	10 (5.5)	10 (5.5)	Gran Canaria island	Caminero *et al*., 2001
1994	29,47	148	12 (8.1)	12 (8.1)	Gran Canaria island	Caminero *et al*., 2001
1995	25,8	110	18 (16.4)	18 (16.4)	Gran Canaria island	Caminero *et al*., 2001
1996	29.0	129	35 (27.1)	35 (27.1)	Gran Canaria island	Caminero *et al*., 2001
1999*	29.3	40	9 (22.5)	nd	Gran Canaria island	not published
2002	20,48	167	19 (11.4)	nd	Gran Canaria island	not published
2003	23,17	165	13 (7.8)	nd	Gran Canaria island	not published
2004	19,1	129	15 (11.6)	nd	Gran Canaria island	not published
2007	17,48	169	37 (21.9)	27 (15.98)	Las Palmas Province	Millan et al., 2012
2008	15,17	151	33 (21.8)	29 (19.2)	Las Palmas Province	Millan et al., 2012
2013	7,18	135	26 (19.2)	24 (17.7)	Canary Islands	This work
2014	7,01	109	27 (24.7)	27 (24.7)	Canary Islands	This work

*(Last quarter).

The strain has been characterized in depth^8,12^. It has a typical RFLP pattern, with 22 IS6110 copies distributed along its genome. It lacks the IS*6110* copy in the NTF locus, which is used as a marker for this lineage. Four different isolates were characterized using WGS, and the SNPs identified revealed it to be a strain belonging to the Beijing subfamily Asia Ancestral 3^4^. The IS*6110* inserted in the Rv2180c gene was determined to be a specific marker of the GC1237 strain, after evaluating a collection of Beijing and not Beijing strains^12^, ruling out that it is a common feature of a particular sublineage. IS*6110*-RFLP was also performed in several isolates from 2007-8 and the patterns obtained by the strain-specific-PCR rapid method and RFLP were fully concordant^8^.

New PCR-based strategies to track specific high-risk strains can now be more easily developed once an individual genetic feature is identified. This is the rationale of the IS*6110* PCR applied in this study. The approach applied in parallel to IS*6110* PCR, was based on a different notion, that is, it targeted strain-specific marker SNPs, which were detected after a thorough analysis of the WGS data of the strain. The robustness of the SNPs targeted by this PCR to be considered as specific for the Beijing-GC1237 strain was demonstrated previously^9^. In brief, the evaluation of its specificity was based on a comparison with the SNPs from a global database including 219 strains representing the geographic and phylogenetic diversity of the *M*. *tuberculosis* complex. To reinforce even more the strength of this specificity analysis we applied a second analysis with a database containing 358 unique Beijing genomes.

The two PCR-based approaches mentioned in the previous paragraphs shared the same potential for speeding up the process of tracking the transmission of high-risk strains or facilitating rapid update of relevant epidemiological problems. Similar ASO-PCRs have been applied to prospectively determine the presence of an MDR *M*. *tuberculosis* strain in Equatorial Guinea^13^ and in retrospective collections and to identify potential secondary cases caused by two XDR Beijing strains imported from Russia to Spain^14^.

The Gran Canaria outbreak provided an excellent opportunity to apply strain-specific PCR-based approaches for rapid updating, whose only requirement is access to the retrospective collections of *M*. *tuberculosis* isolates in the Autonomous Community of Canarias. Inactivated bacteria were delivered, and the analysis was performed directly without purification of DNA, thus saving transport and analysis costs. The performance of the ASO-PCR targeting strain-specific SNPs was more sensitive than the alternative PCR targeting IS*6110*, as can be concluded from the lack of amplification observed with IS*6110*-PCR in the 77 isolates included in one of the deliveries. The quality/quantity of these DNAs may have been affected by a methodological problem. All but 7 of the DNAs were efficiently analyzed using ASO-PCR.

The strain-specific PCR dual approach yielded valuable data. Firstly, the GC1237 strain continues to give cause for concern; 20 years after its appearance, global rates (20.9%) remain high.

The final clarification of whether this situation is due either to a prolonged and persistent transmission of a single strain that was imported in 1993 (with the index case behaving as a superspreader) or, alternatively, to frequent importations of genetically related strains would need systematic WGS analysis, not only for the strains captured in our study but from those collected along 2 decades, to reconstruct the phylogeny of this event. This lies beyond the scope of our study. However, some observations could give us some clues to clarify somehow this point. Firstly, the index case came from Liberia, therefore new independent importations should come from migrants from this same country. In the first article^7^ describing in detail the outbreak, only 4 additional from Liberia were reported, with the vast majority of the cases being Spaniards. The four cases from Liberia were closely related with the index case. In the current update of the situation of the outbreak, all new cases infected by the GC1237 strain were Spaniards, except one case from Sweden. Secondly, the epidemiological investigation of this outbreak in the nineties also found a rapid spread of the outbreak among homeless and migrants, living in poor conditions. All these observations together, minimize the role for independent importations and make recent transmission to be the most likely explanation for the outbreak. Finally, for other transmission events in other countries where it has been described an overlapping between the recent transmission of a strain from a migrant index case who imported a strain, and subsequent independent importations from that strain which is prevalent in their country of origin, a high SNP-based diversity has been described^15,16^. On the contrary, all the GC1237 new cases captured in this study were homogeneous according to the presence in all of them of the set of SNPs selected to target the strain. Again, these observations suggest that the transmission from a single importation event, without additional subsequent importations, is the most likely scenario.

If we focus on the rates recorded in our study for the GC1237 strain in Gran Canaria, the only island with a historic reference, the rate is close to the figures obtained in the most recent snapshot (2008). In addition, for the first time, we had the opportunity to determine whether this problem was restricted to the province where the strain was first identified, Las Palmas de Gran Canaria, or whether it has spread to the entire archipelago. Our data show that the strain is highly prevalent in all of the islands (the lowest value reported is 10.41%) and reaches values even greater than those ever obtained in Gran Canaria (32% on Lanzarote island). The impact of this genotype is uncommon and remarkable.

There are other contexts where specific genotypes have successfully spread in a population. The largest outbreak of tuberculosis in Scandinavia since 1992 was identified in Denmark and ascribed to a single genotype, C2/1112–15. More than a thousand cases infected by this single strain have been identified during the last 23 years^17^. Similar findings were reported in Zaragoza, Spain, where a strain progressed from being low-prevalence to responsible for around 20% of the all tuberculosis cases in the population, with high rates maintained throughout the period^18^.

In other European countries with a low incidence of tuberculosis, which have in common a preponderance of European-American Lineage 4 strains, cross-border and cross-continental movements of people for leisure or work in recent decades have changed socio-epidemiological scenarios. In addition, large-scale migration from countries where tuberculosis is highly endemic^19^ and whose inhabitants, may export strains that were previously unknown in the host population could lead to unexpected rates of transmission.

The marked expansion of the GC1237 strain might be due to either bacterial or epidemiological factors. A case persistently infected by this strain, in a different population, Madrid, did not cause secondary cases^20^, suggesting that epidemiological features might be more likely behind the successful expansion of this strain. An index case with a prolonged diagnostic delay and laringeous involvement (as described in the first studies describing the origin of this outbreak) might have caused a big pool of exposed cases which explains the prolonged presence of the strain.

The figures we report for the Canary Islands warn that we are facing a strain that has not still been efficiently controlled. This genotype has remained present over the years and has completely changed the scenario commonly found in other Western European countries, including the Spanish mainland. Fortunately, in contrast with other Beijing outbreaks, our data revealed a susceptible Beijing pattern, as the resistance phenotype appeared in only 2 cases, thus indicating good therapeutic control of the cases involved. What is surprising is that despite the success of this strain, the incidence of tuberculosis in Canarias is not increasing, indicating that the strain is likely displacing in the population the previously dominant strains. Elimination of this strain in the Canarian population would radically decrease the incidence of tuberculosis in this autonomous community.

Although the strategy applied in or article cannot offer an answer to many of the relevant questions behind this challenging prolonged transmission event, it has helped us to get valuable data to understand the current situation of an uncontrolled outbreak. Using strain-specific tools based on in-depth-knowledge of each strain acquired from genome analysis could enable us to resolve the problem. Strain-specific PCR is an easily implemented and inexpensive tool that can be applied locally in the laboratories managing the outbreak and can constitute a model to be replicated in other settings with challenging transmission events but lacking programs to assure universal molecular/genomic systematic analysis. ASO-PCR has proven useful when applied even directly on respiratory specimens^21^, thus potentially enabling close real-time monitoring of the high-risk GC1237 strain when a new case is diagnosed. The approach we report might constitute the only way to eliminate successful prevalent strains.
